# Independent predictors and risk score for intraprocedural rupture during endovascular treatment of small ruptured intracranial aneurysms (<5 mm)

**DOI:** 10.3389/fneur.2022.923645

**Published:** 2022-08-24

**Authors:** Fei Peng, Xin Feng, Xiaoxin He, Hao Niu, Hong Zhang, Xin Tong, Baorui Zhang, Jiaxiang Xia, Xuge Chen, Boya Xu, Peng Qi, Jun Lu, Daming Wang, Aihua Liu

**Affiliations:** ^1^Department of Interventional Neuroradiology, Beijing Neurosurgical Institute, Beijing Tiantan Hospital, Capital Medical University, Beijing, China; ^2^China National Clinical Research Center for Neurological Diseases, Beijing, China; ^3^Neurosurgery Center, Department of Cerebrovascular Surgery, Engineering Technology Research Center of Education Ministry of China on Diagnosis and Treatment of Cerebrovascular Disease, Zhujiang Hospital, Southern Medical University, Guangzhou, China; ^4^Guangdong Provincial Key Laboratory on Brain Function Repair and Regeneration, Guangdong, China; ^5^Operating Room of Heze Municipal Hospital, Heze City, China; ^6^Department of Neurosurgery, Beijing Hospital, National Center of Gerontology, Beijing, China; ^7^Graduate School of Peking Union Medical College, Beijing, China

**Keywords:** intraprocedural rupture, small, intracranial aneurysms, endovascular treatment, risk score

## Abstract

**Background and purpose:**

Intraprocedural rupture (IPR) is a devastating complication of endovascular treatment (EVT). Small-sized and ruptured aneurysms are independent predictors of IPR, which presents a technical challenge during EVT. We aimed to develop a score to quantify the individual patient risk of IPR in the EVT of small (<5 mm) ruptured aneurysms (SRAs).

**Methods:**

A retrospective review was conducted to interrogate databases prospectively maintained at two academic institutions in China from January 2009 to October 2016. We collected intraoperative angiograms and medical records to identify independent predictors of IPR using univariate and multivariable analyses. A risk score for IPR was derived using multivariable logistic regression analyses.

**Results:**

Of the 290 enrolled patients, IPR occurred in 16 patients (5.5%). The univariate analysis showed that the rate of IPR was significantly higher in patients having aneurysms with a small basal outpouching (SBO), in patients having aneurysms concomitant with adjacent moderate atherosclerotic stenosis (ACAMAS), and in former or current smokers. Multivariate analyses showed that SBO [odds ratio (OR): 3.573; 95% confidence interval (CI): 1.078–11.840; *p* = 0.037], vascular eloquence (VE; OR: 3.780; 95% CI: 1.080–13.224; *p* = 0.037), and ACAMAS (OR: 6.086; 95% CI: 1.768–20.955; *p* = 0.004) were significantly and independently associated with IPR. A three-point risk score (S-V-A) was derived to predict IPR [SBO (yes = 1), VE (yes = 1), and ACAMAS (yes = 1)].

**Conclusions:**

Intraprocedural rupture occurred in 5.5% of the patients during EVT of SRA. SBO, VE, and ACAMAS were independent risk factors of IPR in the EVT of SRA. Based on these variables, the S-V-A score may be useful in predicting IPR daily, but more confirmation studies are required.

## Introduction

Intraprocedural rupture (IPR) during endovascular treatment (EVT) of ruptured intracranial aneurysms (RIAs) is one of the most feared complications with an incidence rate of 1%−8% and a modality rate of up to 40% (1–3). Several studies reported that aneurysm size (<5 mm) and ruptured aneurysm are important independent predictors of IPR (4–6). However, no study has focused on IPR predictors during EVT of small ruptured aneurysms (SRA, <5 mm).

In addition, EVT for SRA has technical challenges, including instability of the distal microcatheter, coil conformability, and the reliability of coil detachment (1, 7), which may lead to IPR. Based on a large number of studies, a common assumption to date was that the size and location of aneurysms, technical aspects, and basal morphology might be associated with the risk of IPR (1, 5, 6). However, those studies did not take into account the heterogeneity of EVT between small and big aneurysms. In this study, we conducted a retrospective analysis of consecutive patients with SRA to identify independent predictors of IPR during EVT.

## Methods

### Patient selection and data acquisition

This retrospective multicenter study was carried out at Beijing Tiantan Hospital and Beijing Hospital. This study included all patients who had a saccular SRA and underwent EVT at the two Chinese stroke centers from January 2009 to October 2016. In this study, the exclusion criteria were (1) patients with fusiform, traumatic, blood blister-like, and dissecting aneurysm; (2) those with aneurysms related to arteriovenous malformation (AVM), arteriovenous fistula (AVF), and moyamoya disease; (3) those with incomplete data (for example, missing digital subtraction angiography (DSA) or progress note); (4) ambiguous information about which aneurysm is ruptured in patients with multiple aneurysms; and (5) patients who did not originally undergo EVT at these two stroke centers.

We obtained these prospectively maintained databases *via* medical records and a detailed inquiry. These data included sex, age, hypertension, diabetes mellitus, dyslipidemia, heart comorbidities, history of smoking, history of drinking, history of subarachnoid hemorrhage (SAH), prehospital delay after SAH, preprocedural delay after SAH and EVT, Hunt and Hess Grade and Fisher Grade at admission, treatment modality, Raymond Scale (RS) score (8), and the modified Rankin Scale (mRS) score at discharge. The RS score demonstrates the occlusion degree of aneurysms at the end of embolization, including complete occlusion (RS1), residual neck (RS2), and residual aneurysm (RS3) (8). We recorded the morbidity and mortality rates associated with the treatment at 1 month after discharge treatment. Morbidity was categorized as an mRS score of 2–5 or at least a one-point increase during hospitalization.

### Imaging of variable definitions

Aneurysm-specific characteristics included aneurysm location, aneurysm size, neck size (9), aspect ratio (AR) (10), and aneurysm shape, aneurysms concomitant with adjacent atherosclerotic stenosis (ACAAS) (2), multiplicity, vascular eloquence (VE) (11), and small basal outpouching (SBO) (12). Aneurysm location was categorized as distal vessels (A2, M2, P2 aneurysms and beyond) (13); VE (parent arteries of IAs <20 mm from the internal carotid artery or located in the first segment of cerebral arteries, e.g., A1, M1, and P1 segments) (11), cerebral bifurcation vessels (internal carotid artery bifurcation and basilar artery) and important vascular branches (posterior inferior cerebellar and anterior choroidal arteries) supporting the brain stem and the basal ganglia area; communicating arteries (anterior and posterior communicating artery); and other areas. SBO was defined as the daughter sac, or bleb, which is located near the base of a ruptured aneurysm (12). ACAAS was defined as aneurysms with proximal parent artery stenosis (2). The degree of stenosis in the proximal parent artery was defined as mild, moderate, and severe ACAAS (i.e., ACAIAS, adjacent moderate atherosclerotic stenosis (ACAMAS), and ACASAS), which correspond to <50, 50–70, and >70%, respectively.

### Endovascular procedures

Each center was equipped with green channels for emergency patients in the acute stage (SAH within 72 h). Patients with SRA underwent endovascular or surgical treatment, which was determined by the neurosurgical team of each center according to the patient's characteristics and aneurysm features. All EVT procedures were performed under general anesthesia and systemic heparinization. After deployment of the first coil, an initial bolus of 50 U/kg heparin was given, and the activated clotting time was maintained at two times the normal range. For wide-neck aneurysms, (14) coiling with a stent or a balloon was performed by interventionalists with 20 years of experience at each center. During the procedure of stent-assisted coiling, patients would be allocated 300 mg clopidogrel and 300 mg aspirin. When IPR occurred, heparin was reversed by protamine sulfate, and rapid coil deposition was always considered until contrast medium extravasation disappeared. Blood pressure would be controlled at baseline. If needed, a balloon would be inflated near the proximal artery in case of massive hemorrhage, and emergency ventricular drainage (EVD) would be performed to reduce intracranial pressure. In case of acute thrombotic events, the glycoprotein Iib/IIIa inhibitor (Tirofifiban, Grand Pharma, Wuhan, China) was used. After the EVT procedure, immediate brain computed tomography (CT) scans were always performed for each patient. Moreover, patients received 75 mg of clopidogrel daily for 1 month and 100 mg of aspirin daily for 5 months.

### IPR definition

Digital subtraction angiography or immediate post-operative CT scans demonstrated that IPR was defined as extravasation of the contrast medium. Two experienced interventionalists (15 and 20 years of experience in neuroradiology, respectively) who were blinded to patients' clinical information reviewed the angiograms. Any disagreements were resolved through negotiation. Considering the missing DSA reports of four cases of IPR, those cases were classified as unclear. We classified the occurrence of IPR into three phases: during aneurysm access, during coil placement, and other phases. The causes of IPR were classified into four categories: coil, microguide wire, microcatheter, and other categories.

### Statistical analysis

Data related to categorical variables were analyzed using Fisher's exact test or Pearson's chi-squared test. Data related to continuous variables were analyzed using two-tailed *t*-tests or the Kruskal–Wallis *H*-test. A univariate analysis was used to compare each variable value between the IPR and non-IPR groups. Variables with a *p-*value ≤ 0.20 would be included in the multivariate analysis. We followed the current guidelines for risk score models (15). A *p*-value ≤ 0.05 (two-tailed) was prospectively considered statistically significant. Statistical analyses were performed with SPSS (version 23.0; IBM Corp., Armonk, NY).

### Risk score derivation

In the final fitted multivariable model, the regression coefficient of each variable was performed to develop the point score to predict the rate of IPR during EVT of SRAs. The final risk score of IPR for each patient was defined as the sum of all points for each variable. The evaluation of the derived score was based on discrimination and calibration. Discrimination, which indicates the ability to discriminate between the IPR and non-IPR groups, was assessed using the C-statistic (areas under receiver operating characteristics curves, AUC; 0.5 indicates no ability and 1.0 indicates perfect ability). Calibration was carried out by comparing the observed probability of IPR against the predicted risk through the Hosmer–Lemeshow goodness-of-fit test (nonsignificant *p-*value of the test indicating a good fit).

## Results

### Study population and baseline characteristics

**Figure 1:**
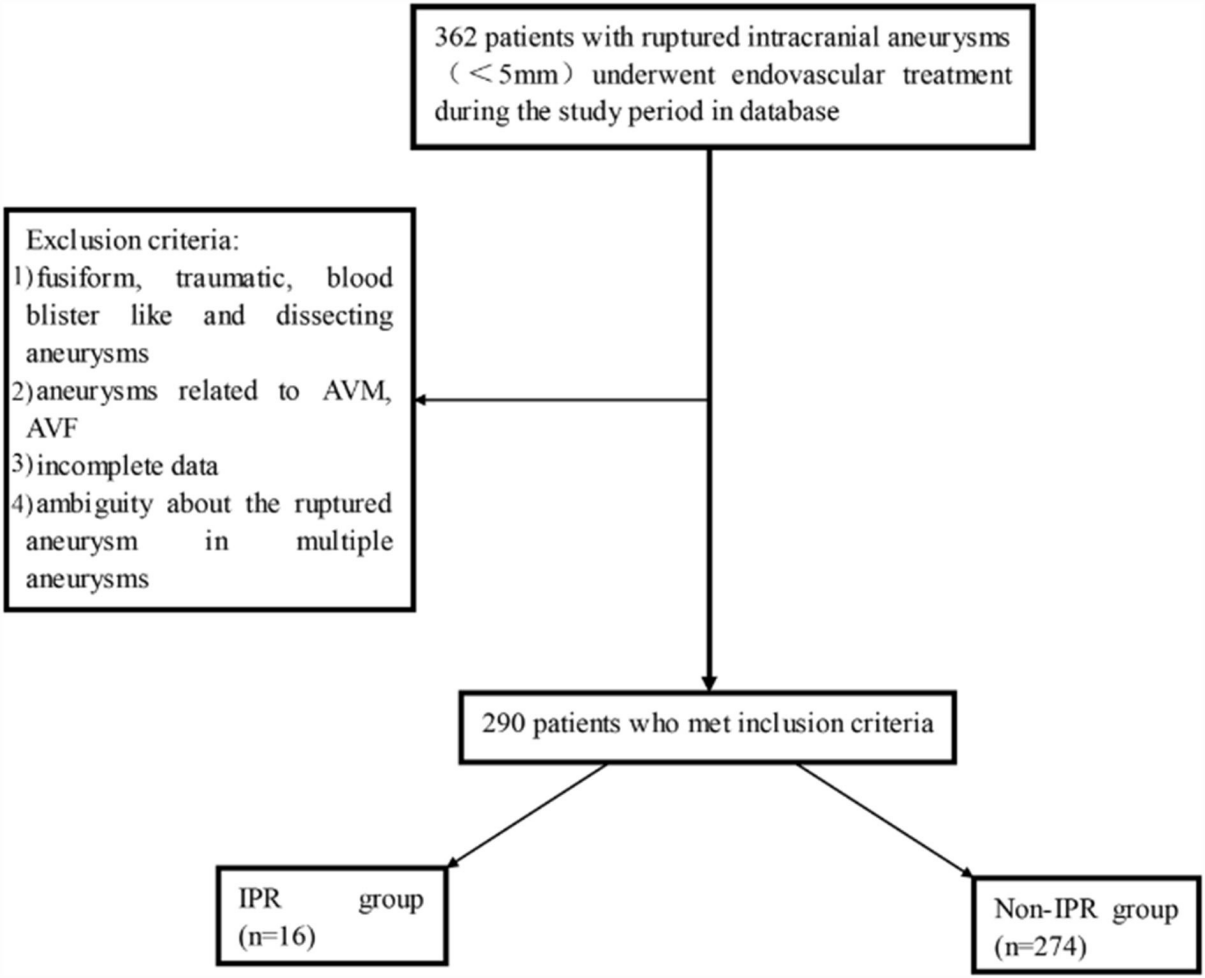
The patient inclusion flowchart. AVM, arteriovenous malformation; AVF, arteriovenous fistula; IPR, intraprocedural rupture.

In total, 362 patients with RIAs (<5 mm) underwent EVT during the study period in the databases. Approximately, 72 patients were excluded, as seen in Figure 1. Finally, 290 patients with SRAs were included. The median age was 53.5 ± 0.69 years (ranging from 14 to 79), and 55.5% were women. The number of patients with SRA located in the VE, distal vessels, communicating arteries, and other arteries was 6 (20.6%), 28 (9.7%), 29 (10.0%), and 173 (59.7%), respectively. The number of patients treated with coiling only, stent-assisted coiling, and balloon-assisted coiling was 191 (65.9%), 83 (28.6%), and 16 (5.5%), respectively. The basic characteristics of patients and aneurysms are presented in Table 1.

**Table 1:** Baseline characteristics of patients and aneurysms in IPR and non-IPR groups.

**Characteristic**	**Total**	**IPR(100%)**	**P-value**
Female	161	10(6.2)	0.615
Age≥ 50 (years)	172	7(4.1)	0.202
Hypertension	177	7(4.0)	0.188
Diabetes mellitus	26	1(3.8)	1.000
Dyslipidemia	87	4(4.6)	0.771
Heart comorbidities	26	0(0.0)	0.376
Smoking atatus			0.031
Never smoking	204	15(7.4)	
Current smoking	79	0(0.0)	
Former smoking	7	1(14.3)	
History of drinking	73	4(5.5)	1.000
History of SAH	31	3(9.7)	0.393
Acute stage	167	6(3.6%)	0.118
Prehospital delay after SAH			0.300
≤ 3 days	165	7(4.2)	
3-14 days	63	3(4.8)	
15-28 days	22	3(13.6)	
>28 days	40	3(7.5)	
Preprocedure dalay after SAH			0.841
≤ 3 days	135	7(5.2)	
3-14 days	73	3(4.1)	
15-28 days	39	3(7.7)	
>28 days	43	3(7.0)	
Hunt Hess Grade			0.726
1-2	244	13(5.3)	
3-5	46	3(6.5)	
Fisher Grade			1.000
1-2	230	13(5.7)	
3-5	60	2(5.0)	
Treatment modality			0.608
Coiling	191	11(5.8)	
Stent-assisted coiling	83	5(6.0)	
Balloon-assisted coiling	16	0(0.0)	
Raymond scale (RS)			0.663
RS1	214	13(6.1)	
RS2	62	2(3.2)	
RS3	14	1(7.1)	
mRS score on discharge			0.162
≤ 2	239	11(4.6)	
>2	49	5(10.2)	
Location of distal vessels	28	2(7.1)	0.659
Communicating arteries	168	9(5.4)	1.000
VE	61	6(9.8)	0.114
neck size			0.374
<4mm	224	11(4.9)	
≥4mm	66	5(7.6)	
Aspect ratio			0.609
<1.3	157	10(6.4)	
≥1.3	133	6(4.5)	
aneurysm size			0.569
≤ 3mm	82	3(3.7)	
3-5mm	208	13(6.3)	
shape of aneurysm			0.183
Lobular	13	0(0.0)	
Regular	135	5(3.7)	
Daughter sac	54	6(11.1)	
Other irregularity	88	5(5.7)	
ACAAS			0.010
ACAIAS	243	10(4.1)	
ACAMAS	38	6(15.8)	
ACASAS	9	0(0.0)	
multiplicity	43	3(7.0)	0.715
SBO	46	6(13.0)	0.027

*VE, vascular eloquence (parent arteries was <20 mm from the internal carotid artery or the first segment of cerebral arteries, eg, A1, M1, P1 segments); SBO, small basal outpouching; ACAAS, aneurysms concomitant with adjacent moderate atherosclerotic stenosis; ACAIAS, the mild of ACAAS; ACAMAS, the moderate of ACAAS; ACASAS, the severe of ACAAS*.

### Characteristics of IPR

The characteristics of IPR are summarized in Table 2. IPR occurred in 16 out of 290 endovascular procedures, and the incidence of IPR was 5.5%. According to the timing of IPR, two cases (12.5%) occurred during aneurysm access, eight cases (50%) occurred during coil placement, and six cases (37.5%) occurred during unclear stages. Additionally, among the causes of IPR, eight were coils, one was a microguide wire, one was a microcatheter, and the other six were other reasons (a case of ruptured aneurysm after stent deployment; a case of ruptured aneurysm from an unclear reason before the microcatheter in position, and four cases with no angiography reports).

**Table 2:** Characteristics, management, and outcomes of IPR (n = 16).

**Characteristics**	**n**	**Incidence (%)**
Timing of perforation
Access	2	12.5
Coils placement	8	50.0
Others	6	37.5
Causes of IPR
Coil	8	50.0
Microguidewire	1	6.3
Microcatheter	1	6.3
Others	6	37.5
Symptoms
Headache	4	25.0
Disturbance of consciousness	3	18.8
Double vision	1	6.3
Limb weakness	2	12.5
Complications
Ischemia	9	56.3
Bleeding	2	12.5
Clinical outcome
Morbidity	8	50.0
Mortality	4	12.5

Among patients with IPR, post-procedural symptoms are shown in Table 2. Eight patients (50%, eight in 16) had morbidity, while four patients (50%, eight in 16) had mortality. The overall morbidity and mortality rates were 38.8 and 2.8%, respectively.

### Univariate analysis and multivariate analyses for risk factors of IPR

The following factors from an univariate analysis were significant (*p* < 0.20) and subsequently included in the multivariable analyses: SBO (*p* = 0.027), the shape of an aneurysm (*p* = 0.183), acute stage (*p* = 0.118), smoking status (*p* = 0.031), ACAAS (*p* = 0.010), hypertension (*p* = 0.188), and VE (*p* = 0.114). Multivariate analyses showed that aneurysms with an SBO [odds ratio (OR): 3.573; 95% confidence interval (CI): 1.078–11.840; *p* = 0.037], with ACAMAS (OR: 6.086; 95% CI: 1.768–20.955; *p* = 0.004), and in VE (OR: 3.780; 95% CI: 1.080–13.224; *p* = 0.037) were independently associated with IPR, which were entered into the finally adjusted multivariable logistic regression model (Table 3).

**Table 3:** Multivariate analysis of risk factors for intraprocedural rupture.

**Variable**	**OR(95%CI)**	***P value***
Hypertention	0.308(0.095-0.996)	0.049
Smoking	2.797(0.277-28.262)	0.383
VE	3.780(1.080-13.224)	0.037
SBO	3.573(1.078-11.840)	0.037
ACAMAS	6.086(1.768-20.955)	0.004

*SBO, small basal outpouching; ACAMAS, aneurysms concomitant with adjacent moderate atherosclerotic stenosis*.

### Risk score derivation

**Figure 2:**
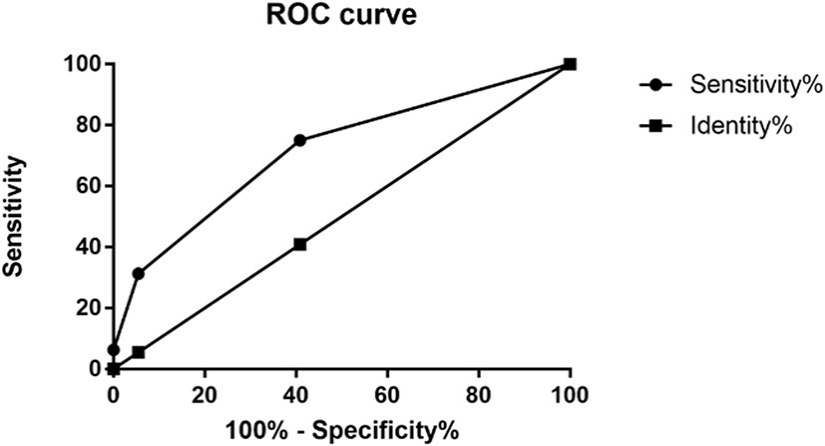
Receiver operating characteristic (ROC) curves for the IPR score. The AUC value was 0.716 (95% CI: 0.58–0.86) for this model. AUC, area under the curve; CI, confidence interval. IPR, intraprocedural rupture.

**Figure 3:**
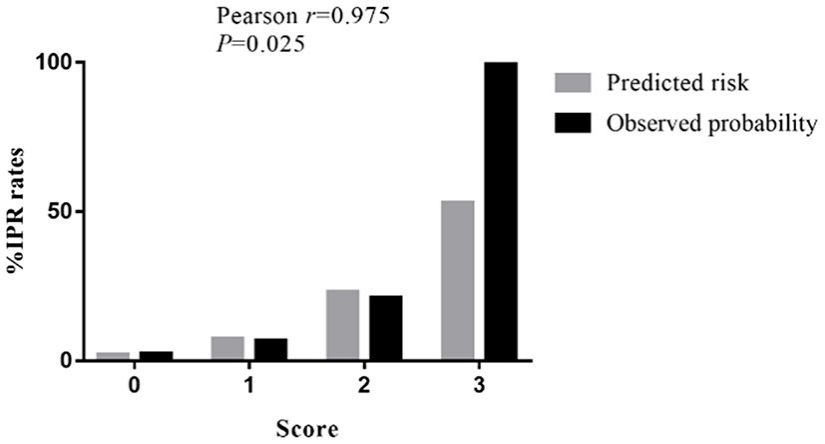
Rates of IPR by the clinical score for patients with SRAs. The predicted rates of patients with SRAs with a score of 0, 1, 2, and 3 have IPR rates of 2.1, 7.4, 23.0, and 53.0%, respectively. The observed rates of patients with SRAs with a score of 0, 1, 2, and 3 have IPR rates of 2.4, 6.7, 21.1, and 100%, respectively. IPR, intraprocedural rupture; SRAs, small ruptured aneurysms.

According to the multivariate analyses, a three-point IPR score was derived (Table 4). The discriminative power of the IPR score was statistically significant (AUC: 0.716; CI: 0.575–0.856; *p* = 0.004), as seen in Figure 2. The Hosmer–Lemeshow χ^2^ statistics were 0.159 (*p* = 0.650) in the cohorts, indicating good calibration. Figure 3 reports observed and predicted probabilities of the IPR rate according to the IPR risk score. The predicted IPR rates for patients with a score of 0, 1, 2, and 3 were 2.1, 7.4, 23.0, and 53.0% respectively, which were significantly correlated with their actually observed IPR rates (2.4, 6.7, 21.1, and 100% respectively; Pearson *r* = 0.975, *p* = 0.025; Figure 3).

**Table 4:** Risk score (S-V-A score, 0-3 points) for IPR.

**Clinical variables**	**Assigned points**
SBO
No	0
Yes	1
VE involvement
No	0
Yes	1
ACAMAS
No	0
Yes	1

## Discussion

The rate of IPR in patients with ruptured aneurysms ranges from 2.73 to 9.52% (5, 16). We observed a comparable rate of 5.5% in the present study. Smaller aneurysm size (<5 mm) and ruptured IAs were reported as independent predictors of IPR. However, very few studies were designed to identify independent predictors of IPR during the coiling of small and ruptured IAs (1, 3, 17). In this study, we found that SBO, VE, and ACAMAS were independent risk factors of IPR in the EVT of SRA.

Small basal outpouching (i.e., a daughter sac or bleb that is located near the base of a ruptured aneurysm) is a common morphological configuration in cases of basal rupture. Park et al. (18) reported that the incidence of SBO in ruptured aneurysms was 8.7%, and in some cases, the basal rupture could be induced by the SBO. Kang et al. (12) suggested that the chance of IPR could increase when the coil or microcatheter was placed near the SBO. The presence of the microcatheter and the microguide wire would likely cause repeated wear and tear of the SBO during the procedure, which also increases the risk of IPR. Similarly, in our study, the risk of IPR in aneurysms with an SBO was three times more than that of an aneurysm without SBO.

Several previous studies reported a case series of IAs concomitant with ACAAS (2, 19, 20). In this study, of 16 IPR cases, it was found that six (37.5%) had ACAAS and all of them had a moderate degree. It was reported that moderate or severe stenosis adjacent to the aneurysm may increase the risk of aneurysm rupture due to pressure changes caused by balloon or stent implantation (19). One possible explanation is that atherosclerosis could increase vascular susceptibility to mechanical injury (21), and the rebleeding event may be triggered by the coil implanted near the aneurysm neck, which was proximal to the stenosis. However, of the nine cases with ACASAS, none of them had IPR. One possible explanation is that the occlusion rate of was higher in SRAs with ACAMAS (92.1%) than SRAs with ACASAS (55.6%). Moreover, we should be more careful in treating proximal severe stenosis cases and use coil occlusion as much as possible to avoid the occurrence of a thromboembolic event, which may further reduce the contact with the aneurysmal wall and decrease the risk of IPR.

VE, which is demonstrated as the arterial segments often having iatrogenic complications (i.e., occlusion or injury), was first used to assess the curative risk of EVT for AVM (11). Here, VE is regarded as the parent vessel close to the internal carotid artery or the first segment of cerebral arteries. As reported in the A1 segment of the internal carotid artery bifurcation, EVT of small aneurysms faces a great challenge (17). In this study, six IPR cases (9.8%) occurred in the VE group, which was more than two times that in the non-VE group (4.4%). Among the six IPR cases, four cases (66.7%) occurred in the procedure of coiling. For small-sized aneurysms, the incidence of “close contact” between the coils and the perforators or vessel branches may increase, which may further increase the chance of IPR (17).

It is therefore beneficial to establish a risk score to identify patients who are at high risk of IPR before EVT decision for SRAs. Based on the above three significant and important factors, we established this S-V-A score system. Our receiver operating characteristic (ROC) analysis demonstrated that the application of the endovascular S-V-A score provides good discriminatory power to evaluate IPR rates (AUC = 0.716). The Hosmer–Lemeshow χ^2^ statistics were 0.159 (*p* = 0.650) across cohorts, indicating good calibration. The analysis of predicted risk and observed probability of IPR demonstrated that the predicted IPR rate was significantly correlated with the observed IPR rate (Pearson *r* = 0.975; *p* = 0.025; Figure 3). This score would be helpful to identify patients with a high risk of IPR, thereby taking protective interventions before EVT.

Notably, 50% (eight of 16) of IPR events occurred because of the attempt to reach a complete occlusion. In the procedure of coiling, as the degree of embolization increases, the operation time and the difficulty of operation also increase, which therefore increases the likelihood of IPR. Several factors such as anterior communicating artery (ACoA), posterior communicating arteries (PCoA), history of SAH, (17, 22) coronary artery disease, and dyslipidemia were reported to be the risk factors of IPR in previous studies (4). However, in our study, these risk factors were nonsignificant. Vessel wall disease may be responsible for IPR as it is reported that IPR may be due to vascular rupture (22). However, not all the patients performed high-resolution magnetic resonance imaging (HR-MRI) prior to EVT. Moreover, the vessel wall abnormality was also rarely considered in several previous studies and clinical practice. So, we suggest that there is a need for prospective cohort studies in the future to investigate the association between IPR and vessel wall abnormality demonstrated by HR-MRI or other photographic methods. Many studies found that aneurysms (≤ 3 mm) were associated with an increased incidence of IPR (3, 17). In our study, three cases (3.7%) in the ≤ 3 mm group and 11 cases (5.3%) in the 3–5-mm group had IPR. The IPR rate in the 3-mm group was lower than that in the 3–5-mm group (*p* = 0.569), which was different from their results. The reason may be the limited sample in our study and the heterogeneity of operative techniques in different operators.

### Strengths and limitations

This is the first study to develop a simple scoring system to predict IPR risk in the EVT of SRA. To avoid selection bias, we included information from two major stroke centers in China. Moreover, we included new risk factors, including prehospital delay after SAH and preprocedural delay after SAH, SBO, VE, and ACAAS. In the meantime, we also acknowledge some potential limitations. First, the study has 16 IPR events and offers many predictive variables in the model for this number of events (general rule one variable for 10 events). Second, our study can be limited due to its retrospective nature because data were prospectively recorded with limited data size. Third, the S-V-A score system is designed for SRAs (<5 mm), which may not fit for all ruptured aneurysms. Fourth, some potential risk factors are missing such as softness and the shape of the coils used, vascular wall disease with high-resolution MRI, and stent design and deployment technique. Thus, the S-V-A score requires external validation using a large series in other centers or countries.

## Conclusions

In this study, SBO, VE, and ACAMAS were independent risk factors of IPR in EVT of SRA. This indicates that SBO and ACAMAS are important morphological risk factors for predicting a higher risk of IPR, but more confirmation studies on these results are required. In addition, the S-V-A score may be useful to predict the risk of IPR based on variables used in daily practice.

## Data availability statement

The raw data supporting the conclusions of this article will be made available by the authors, without undue reservation.

## Ethics statement

The studies involving human participants were reviewed and approved by the Ethics Committee of Beijing Tiantan Hospital, Capital Medical University. The patients/participants provided their written informed consent to participate in this study. Written informed consent was obtained from the individual(s) for the publication of any potentially identifiable images or data included in this article.

## Author contributions

FP drafted the manuscript for intellectual content. XF and XH designed the study and analyzed and interpreted the data. HN, HZ, XT, BZ, JX, XC, BX, PQ, and JL collected the data in each center. DW and AL strictly revised this manuscript. All authors contributed to the article and approved the submitted version.

## Funding

This work was supported by the Natural Science Foundation of China (Nos. 82171290 and 81771233), Beijing Municipal Administration of Hospitals' Ascent Plan (DFL20190501), the Natural Science Foundation of Beijing, China (Nos. L192013, 22G10396, 7222050, and 7142032), and Specific Research Projects for Capital Health Development (2018-2-2041).

## Conflict of interest

The authors declare that the research was conducted in the absence of any commercial or financial relationships that could be construed as a potential conflict of interest.

## Publisher's note

All claims expressed in this article are solely those of the authors and do not necessarily represent those of their affiliated organizations, or those of the publisher, the editors and the reviewers. Any product that may be evaluated in this article, or claim that may be made by its manufacturer, is not guaranteed or endorsed by the publisher.
